# Acidification decreases microbial community diversity in the Salish Sea, a region with naturally high pCO_2_

**DOI:** 10.1371/journal.pone.0241183

**Published:** 2020-10-28

**Authors:** Lisa T. Crummett

**Affiliations:** Life Sciences, Soka University of America, Aliso Viejo, California, United States of America; CSIR-National Institute of Oceanography, INDIA

## Abstract

Most literature exploring the biological effects of ocean acidification (OA) has focused on macroscopic organisms and far less is known about how marine microbial communities will respond. Studies of OA and microbial community composition and diversity have examined communities from a limited number of ocean regions where the ambient pH is near or above the global average. At San Juan Island (Salish Sea), a region that experiences naturally low pH (average = 7.8), the picoplankton (cell diameter is 0.2–2μm) community was predicted to show no response to experimental acidification in a three-week mesocosm experiment. Filtered seawater mesocosms were maintained via semicontinuous culturing. Three control mesocosms were maintained at pH 8.05 and three acidified mesocosms were maintained at pH 7.60. Total bacteria was quantified daily with a flow cytometer. Microbial communities were sampled every two days via filtration followed by DNA extraction, 16S rRNA amplification, and MiSeq sequencing. There was no significant difference in total bacteria between pH treatments throughout the experiment. Acidification significantly reduced Shannon’s diversity over time. During the final week of the experiment, acidification resulted in a significant decrease in Shannon’s diversity, Faith’s phylogenetic distance, and Pielous’s Evenness. ANCOM results revealed four bacterial ASVs (amplicon sequence variants), in families Flavobaceriaceae and Hyphomonadaceae that significantly decreased in relative frequency under acidification and two bacterial ASVs, in families Flavobacteriaceae and Alteromonadaceae, that significantly increased under acidification. This is the first OA study on the microbial community of the Salish Sea, a nutrient rich, low pH region, and the first of its kind to report a decrease in both picoplankton richness and evenness with acidification. These findings demonstrate that marine microbial communities that naturally experience acidic conditions are still sensitive to acidification.

## Introduction

Ocean acidification (OA) is caused by an increase in atmospheric CO_2_, which results in an increase in pCO_2_ in the ocean [1]. Naturally, CO_2_ combines with ocean water to form carbonic acid and this reduces the pH of ocean water [2]. Acidification is a major concern, as it is projected to impact all ocean regions [3, 4] and affect a wide variety of marine life [5–7]. Decreased survival, calcification, growth, development, and abundance has been observed from a wide variety of organisms [7] in response to a decrease in pH associated with year 2100 scenarios [8]. However, most efforts have focused on how metazoans will respond to OA, with comparatively fewer OA studies involving marine microbes, despite the fact that heterotrophic and autotrophic bacteria, as well as microzooplankton, play very important roles in the marine food web; as they are major consumers and producers of organic carbon [9–11].

Studies examining microbial community composition and diversity in response to OA have been limited and have yielded inconsistent results [12–17]. This inconsistency may be associated with variation in direct effects (physiological and biochemical responses) to OA but it may also stem from variation in nutrient levels of the source waters or from adding nutrients at different stages of the experiment [18, 19]. In addition, microbial species may increase or decrease in abundance under OA as a result of shifts in competition and/or predation, which will depend on which microbial community members are present [20] in different ocean regions. Thus, it is important to conduct OA experiments on microbial community composition and diversity with natural microbial communities from ocean regions around the globe that vary in nutrient levels [18, 21]. It is also important to conduct these experiments in regions that vary in ambient sea surface pH. The ambient pH could act as a selection pressure that determines how sensitive microbial communities are to OA. Sensitivity to any disturbance is associated with the physiological plasticity and adaptation among microbial community members [22]. Thus far, OA studies on microbial composition and/or diversity have been conducted in a limited number of ocean regions where sea surface pH is near or even greater than the global average [13–17]. In a marine environment that naturally experiences acidified conditions and regular, significant fluctuations in pCO_2_ and pH, experimental acidification may have no effect on microbial community composition or species diversity.

The San Juan Islands (USA; collectively known as the Salish Sea) are bathed in upwelled acidified (pH < 7.75) seawater originating from the California Undercurrent (CU) [23, 24]. Surface waters in the Strait of Juan de Fuca (surrounding the San Juan Islands) have also become more acidic (average pH decline = -0.018 per year) over the past few decades [25]. Failures at the Pacific Northwest oyster larvae hatcheries have been associated with upwelled acidic seawater between 2005 and 2009 [26]. Potential drivers of ocean acidification in this region include upwelling of acidified water, increased eutrophication, increased freshwater input, increased input of dissolved organic carbon, and acidic precipitation [25, 27]. Various levels of input from these drivers contribute to a broad range of pH variability in this inland sea over geographic space [28] and across seasons [23, 29]. At a single location within the Salish Sea, San Juan Island, pCO_2_ and pH were regularly measured over two years with an average pCO_2_ of 683 ± 115 μatm and an average pH of 7.8 ± 0.06, which is well below the global average of pH 8.1 [23]. During that two-year time frame, those values dropped to as low as 400 μatm pCO_2_ and pH 7.6 [23]. Surface water pH values in this region reach their highest levels (> 8.0) in the spring and summer months when salinity drops as a result of freshwater influxes [30]. Naturally reoccurring high pCO_2_ and low pH in the Salish Sea could pose a significant selection pressure on microbial cell physiology (proton pumps, membrane transporters, RubisCo specificity, CCM efficiency, etc.) resulting in a microbial community that may already be adapted to acidified conditions. I predict that the picoplankton community of the Salish Sea will be resistant to OA during a three-week mesocosm experiment using semi-continuous cultures (no nutrient additions). This duration was shown to be sufficient for detection of community-level responses to OA [31]. In this study, I test the null hypothesis of no effect of OA on (1) microbial species diversity, and (2) microbial community composition in a sample taken from the Salish Sea.

## Methods

### Mesocosm experiment

**Fig 1:**
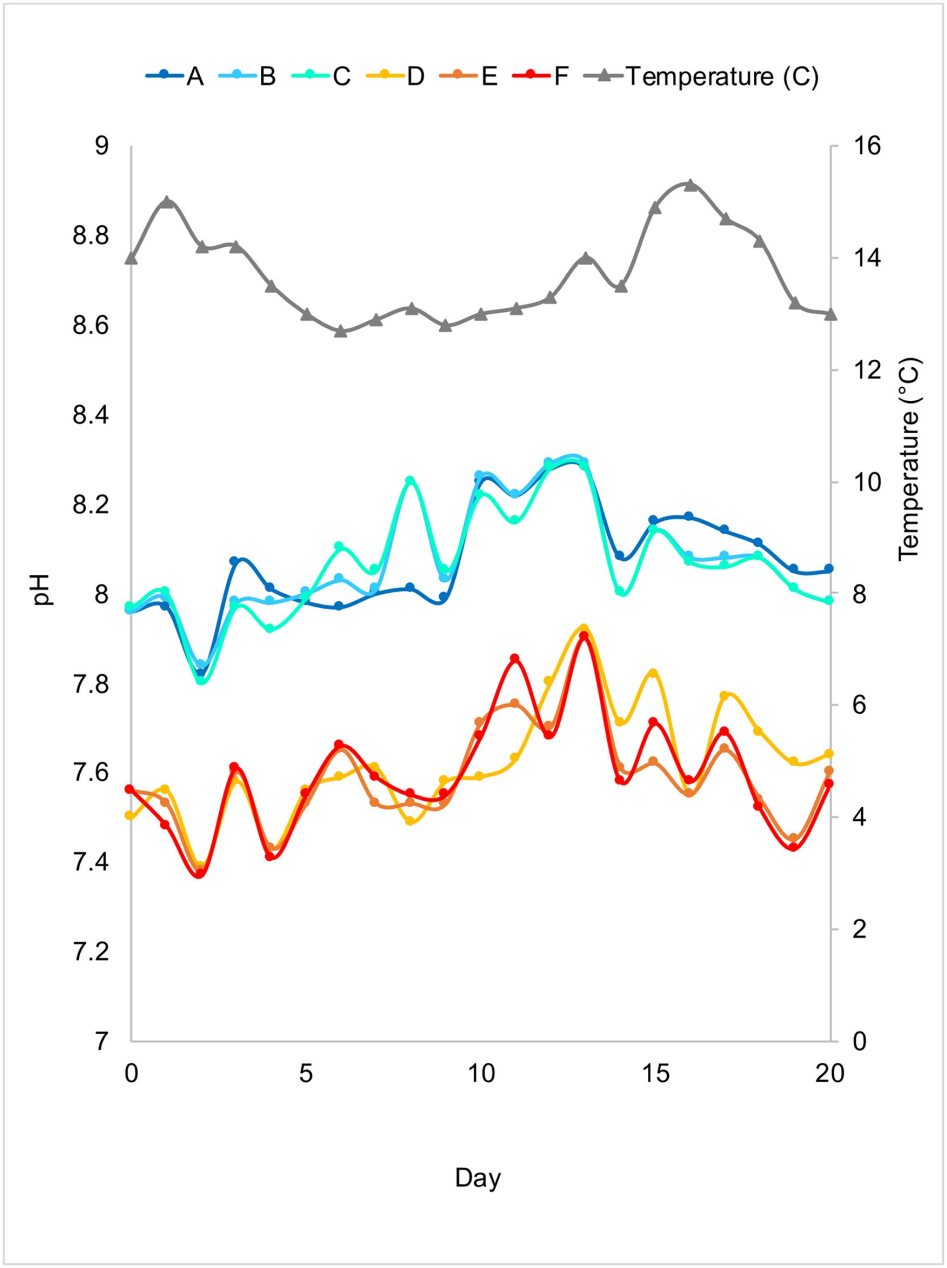
Observed pH and temperature of mesocosms during a 21-day experiment. Replicates A-C are the control mesocosms and replicates D-F are the acidified mesocosms.

A 21-day mesocosm experiment was performed from July 15^th^ 2016 to August 4^th^ 2016 at Friday Harbor Laboratory on San Juan Island off the coast of Washington (USA). Semi-continuous batch cultures [32] were maintained whereby 50% of the seawater within a mesocosm was replaced with fresh 0.2 μm-filtered seawater every two days to avoid potential confounding effects of nutrient additions [18, 19, 33]. Six four-liter glass Erlenmeyer flasks (acid-washed) were filled with three liters of 2.7 μm-filtered sea water from Cantilever Point (48°32'45.6"N 123°00'26.9"W) at a depth of two meters below mean lower low water. The seawater had an initial pH of 7.95 and it was put through a 2.7 μm filter to exclude larger algae and more importantly, to exclude microzooplankton grazers. Each mesocosm flask was placed into a five-gallon bucket that was filled with deionized water up to the height of the seawater in each flask. Each of the six buckets was placed into an open Plexiglas water bath with ambient-temperature sea water continuously flowing through it. The temperature of the water bath ranged from 12.7 °C to 15.3 °C (Fig 1). Three control mesocosm flasks containing seawater were kept near the ambient pH at the time of the experiment, which is also the average global ocean pH (8.05 ± 0.05 standard error), by bubbling ambient air into the seawater at a rate of 5.5 L/min for one hour twice a day at 9:30 am and 9:30 pm (Fig 1). Seawater samples collected from the San Juan Channel during the time of the experiment (July 26 and July 28, 2016) had a pH of 7.92 and 8.01 respectively. Thus, the pH of the control flasks was representative of the ambient seawater during the time of the experiment and similar pH values have been observed in these waters during the summer and spring when there is a drop in salinity [23, 30]. Three treatment mesocosm flasks were kept at a lower pH range of 7.60 ± 0.05 standard error by bubbling in a mixture of ambient air (5.5 L/min) and CO_2_ (5.0 mL/min) for one hour twice a day at 9:30 am and 9:30 pm (Fig 1). A target pH of 7.60 was chosen for the low-pH (acidified) treatment because if current trends in CO_2_ emissions continue, the pH of the surface ocean could decrease by another 0.3 to 0.4 units by 2100 [8] and further, a pH of 7.60 is already experienced naturally in some of the areas of the Salish Sea, and occasionally, off San Juan Island [23].

Mass flow controllers (Aalborg GFC, New York) were used to control the flow rate of air/gas into the mesocosm flasks during bubbling. The pH of the seawater in the mesocosms was measured with a Honeywell Durafet pH electrode that was connected to a Honeywell UDA2182 process controller. Each mesocosm flask was sealed with parafilm to minimize exchange of gases between the headspace of the flask and the ambient air during the interim between bubbling twice a day. This method was shown to be effective at maintaining the target pH of the sea water throughout the experiment (Fig 1). A sheet of thick plastic was placed over the top of the water bath that reduced ambient sunlight by 50%, as measured with a QSL-2100 radiometer (Biospherical Instruments, San Diego). During the 50% water changes, 1.5 L of the discarded water from each mesocosm was filtered through a 0.2μm-Sterivex filter to collect a sample of the microbial community for DNA extraction.

In addition to sampling the microbial communities of the six mesocosms every two days for three weeks, I also sampled the microbial community from (1) the seawater sample that was used to initiate the mesocosm experiment before it was filtered through a 2.7 μm filter, and (2) a seawater sample that was collected from San Juan Channel offshore from the Friday Harbor Marine Laboratory (48°34’24” N, 123°0’21”W). These samples were compared to the “day 0” mesocosm sample to ensure that the microbial community composition was equivalent. No permits were required for this study, which complied with all relevant regulations.

### Flow cytometry

Each morning, for 21 days, a 1-mL sample was taken from each mesocosm to measure total abundance of bacteria using a Novocyte flow cytometer (ACEA Biosciences Inc. San Diego) with a red and blue dual-laser system. Each sample was fixed with glutaraldehyde (0.1% final concentration) for 15 minutes at room temperature, then diluted 1:10 with 0.2 μm-filtered TE buffer, and finally, each sample was stained with SYBR Gold nucleic acid stain (final concentration 1:40,000) for 15 minutes at room temperature. These fixed, SYBR-stained samples were used to quantify the total bacteria in each sample.

A “repeated measures” general linear model was performed with IBM SPSS Statistics for Windows, version 24 (IBM Corp., Armonk, NY, USA) with total bacteria per μl as the dependent variable, pH as the fixed factor, and day (21 levels) as the within-subject (repeated measures) variable. A type-III sum of squares design was used.

### DNA extraction, 16S rRNA amplification, and MiSeq sequencing

To each Sterivex filter containing bacteria from the mesocosms, 1620 μL of lysis buffer was added. The lysis buffer included 5.84 g NaCl, 64.18 g sucrose, 12.5 mL Tris HCl 1M, and 10 mL EDTA 0.5M dissolved in 150 mL of ultrapure (Milli-Q) water. Sterivex filters were sealed and frozen at -20°C until processed for DNA extraction (within one month). Sterivex filters were thawed at room temperature and microbial DNA was extracted and cleaned in an overnight procedure (S1 File) using lysozyme, proteinase K, sodium acetate, isopropanol, and a Zymo DNA Clean & Concentrate kit (Zymo Inc. Irvine). Illumina 16S metagenomic sequencing library prep protocol [34] was followed with the recommended 16S V3 and V4 primers (forward primer: 5' -TCGTCGGCAGCGTCAGATGTGTATAAGAGACAGCCTACGGGNGGCWGCAG– 3', reverse primer: 5'–GTCTCGTGGGCTCGGAGATGTGTATAAGAGACAG– 3').

Indexed samples were sent to the Institute for Integrative Genome Biology at the University of California, Riverside for sequencing with a MiSeq platform using paired-end 300 bp reads.

### Microbial community analyses with Qiime 2

Qiime2-2019.4 software [35] was used to perform statistical analyses on paired end 300 bp reads of the V3 and V4 regions of the 16S rRNA gene. All of the sequence read data from the mesocosm experiment and the two additional samples (unfiltered day 0 sample and San Juan Channel sample) were submitted to NCBI’s sequence read archive (BioProject/ SRA accession number PRJNA591203). Raw reads were demultiplexed and DADA2 [36] was used to denoise sequence data and filter out chimeric sequences and phiX reads. The primers sequences were trimmed from the ends of the reads and the length of the reads were truncated to 250 bp to maintain quality scores at a minimum of 27. The DADA2 denoise algorithm identifies unique sequences and generates 100% ASVs (amplicon sequence variants). An additional chimera removal step was performed using the “vsearch uchime-denovo” command [37]. Reads were aligned using MAFFT, a multiple sequence alignment program [38], and then, the alignment was masked (filtered) to remove ambiguously aligned regions. FastTree [39] was used to generate a guide phylogenetic tree (midpoint rooted) from the masked alignment for diversity analyses.

Alpha and beta diversity analyses were performed on rarified sequence data. Samples were rarified to a depth of 15234 sequences, which was the maximum depth at which all samples had an equal number of sequences. The practice of rarefaction has recently been criticized for yielding biased results when taxa are unobserved [40]. However, rarefaction plots showed that ASV diversity, as well as various measures of alpha diversity, leveled off (the slope approached zero) at the designated sampling depth of 15234 sequences, demonstrating that additional features (taxa) would not likely be observed beyond that sampling depth (S1 Fig). For alpha diversity, Shannon’s diversity index (quantitative measure of species richness), Faith’s phylogenetic distance (qualitative measure of community richness) and Pielous’s evenness (community evenness) were compared between pH treatments using a Kruskall-Wallis H-test during each of the three experimental time periods (day 2–6, 8–12, and 14–20). In addition to binning alpha diversity measures into three time periods, alpha diversity measures were compared between pH treatments continuously, over the entire 21-day experiment, using linear mixed effects (LME) models.

For beta diversity, weighted UniFrac distance (a quantitative measure of community dissimilarity that incorporates phylogenetic distance and taxon abundance) was calculated among pH treatments specifically during day 14–20 based on the alpha diversity results. A pairwise PERMANOVA analysis was used to determine if the weighted UniFrac distances of samples in different pH treatments were significantly greater than the weighted UniFrac distances of samples within the same pH treatment during day 14–20. Principle coordinates analysis (PCoA) plots were generated from the weighted UniFrac distances for principle coordinate 1 vs. experiment day and for principle coordinate 2 vs. experiment day.

Finally, the taxonomic composition of the samples from each of the pH treatments were examined using a Naive Bayes classifier [41] that was built into Qiime2 and was trained on the Greengenes 13_8 99% OTUs reference database [42] for which the sequences had been trimmed to only include the V3-V4 region of 16S rRNA bound by the primers that were used. An analysis of composition of microbiomes (ANCOM) [43] was performed at a level-6 taxonomic depth (genus level) among samples with different pH treatments during day 14–20. Those ASVs with significantly different abundance among samples from different pH treatments were assigned to a taxon using the Greengenes 13_8 99% OTU database [42] and secondarily, NCBI’s [44] 16S rRNA database (July 2019).

## Results

### Total bacteria

**Fig 2:**
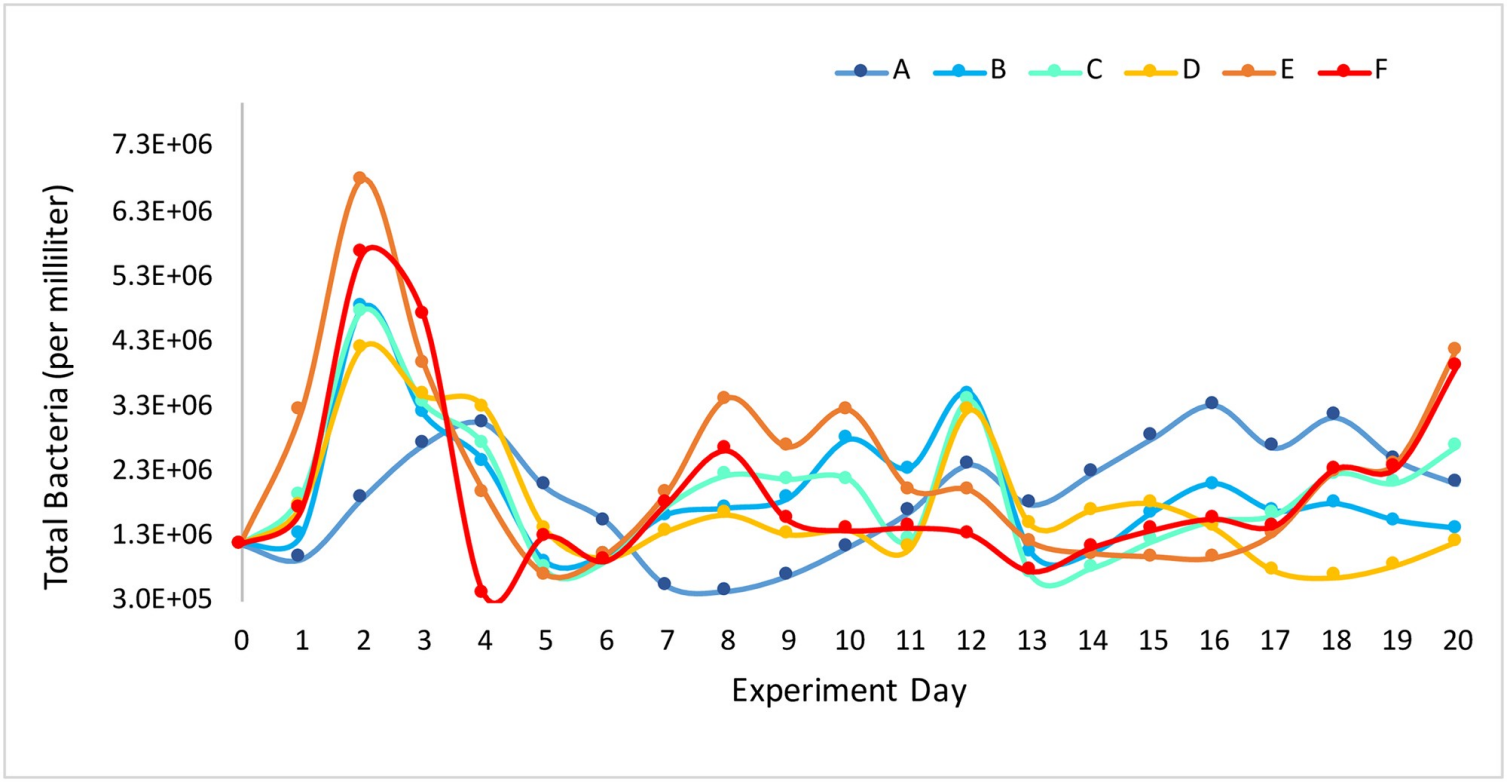
Total bacteria observed each day during a 21-day experiment. Mesocoms A-C were maintained at pH 8.0 and mesocosms D-F were maintained at pH 7.6.

The total number of bacteria present in the mesocosms ranged between 4.2 x 10^5^ and 7.1 x 10^6^ cells per mL throughout the 21-day experiment with a grand mean of 2.0 x 10^6^ ± 1.1 x 10^6^ cells per mL (Fig 2). There was no consistent pattern in bacterial abundance in association with pH treatment as a main effect (*F* = 0.161, *p* = 0.715) or with pH treatment * day as an interaction (*F* = 1.445, *p* = 0.127) throughout the 21-day experiment (Fig 2).

### Alpha diversity decreased with acidification

**Fig 3:**
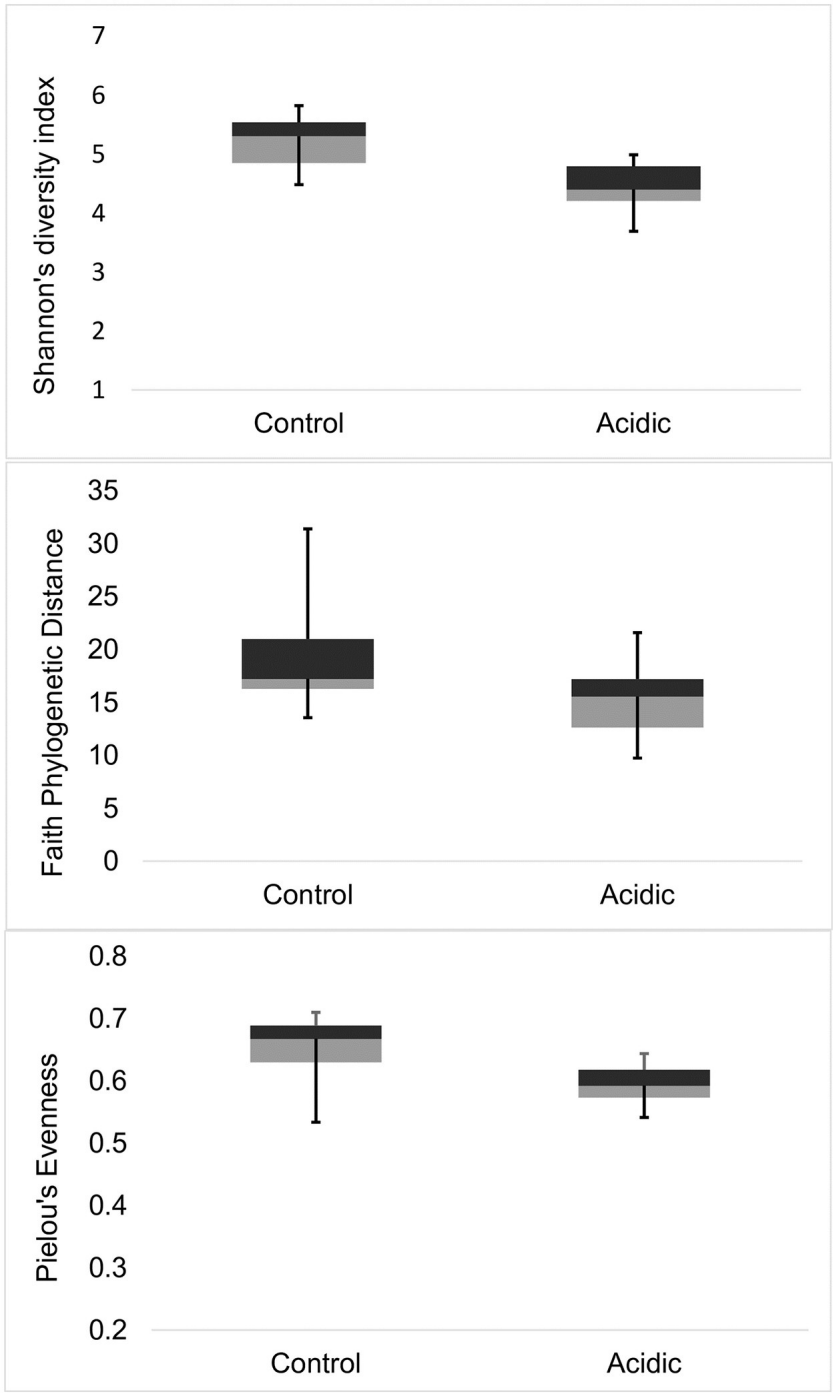
Alpha diversity measures during day 14–20 between two pH treatments. The control mesocosms were maintained at pH 8.0 and the acidic mesocosms were maintained at pH 7.6.

All of the alpha diversity measures were significantly lower in acidified treatments compared to control treatments during the final week of the experiment (day 14–20), including Faith’s phylogenetic distance (Mean ± SD = 19.026 ± 4.900 for the controls versus 14.714 ± 4.205 for the acidified treatment; Kruskal-Wallis *H* = 3.853 and *p* = 0.049), Shannon’s diversity (Mean ± SD = 5.106 ± 0.660 for the controls versus 4.414 ± 0.471 for the acidified treatment; Kruskal-Wallis *H* = 6.163 and *p* = 0.013), and Pielous’s evenness (Mean ± SD = 0.642 ± 0.593 for the controls versus 0.593 ± 0.037 for the acidified treatment; Kruskal-Wallis *H* = 5.333 and *p* = 0.021) (Fig 3). During the earlier phases of the experiment, there was no significant difference between pH treatments in any of the alpha diversity measures including Shannon’s diversity (day 2–6: *p* = 0.102; day 8–12: *p* = 0.757), Faith phylogenetic distance (day 2–6: *p* = 0.508; day 8–12: *p* = 0.964), and Pielou’s evenness (day 2–6: *p* = 0.122; day 8–12: *p* = 0.566).

**Fig 4:**
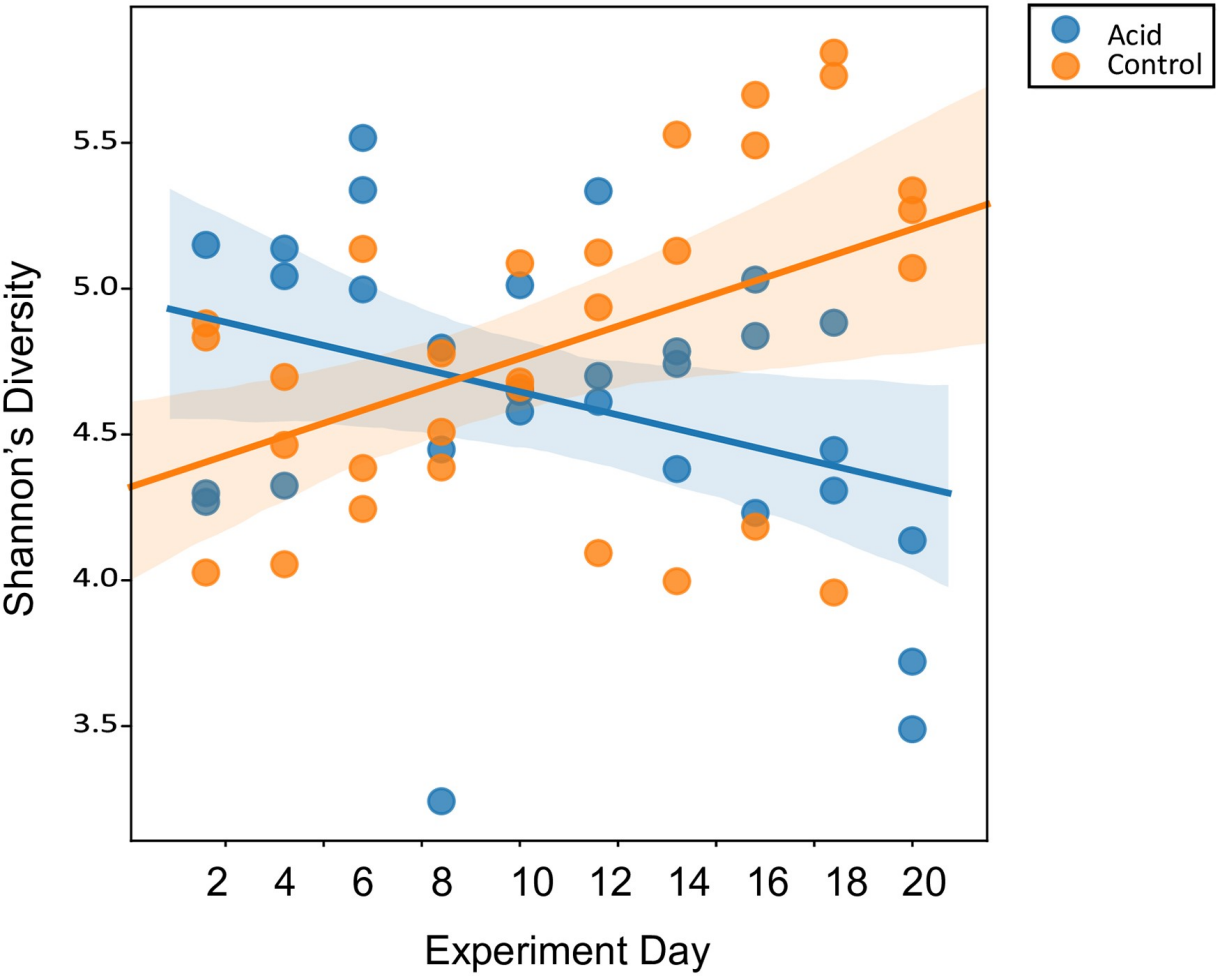
Scatter plot of Shannon’s diversity versus experiment day with linear regression lines and 95% confidence intervals for pH treatment as a fixed effect. A linear mixed effects model compared the two regression lines.

Linear mixed effects models that examined alpha diversity measures in response to pH treatment throughout the entire 21-day experiment, showed that, as a main effect, pH was a significant predictor of Shannon’s diversity (coefficient = -0.648 ± 0.340 standard error, *p* = 0.05; Fig 4) as was experiment day (coefficient = -0.032 ± 0.014 standard error, *p* = 0.02; Fig 4) and the interaction between pH and experiment day (coefficient = 0.076 ± 0.020 standard error, *p* < 0.00). As a main effect, pH was not a significant predictor of Faith’s phylogenetic distance (*p* = 0.44) or Pielou’s evenness (*p* = 0.17).

### Beta diversity between pH treatments was significantly greater than within pH treatments

**Fig 5:**
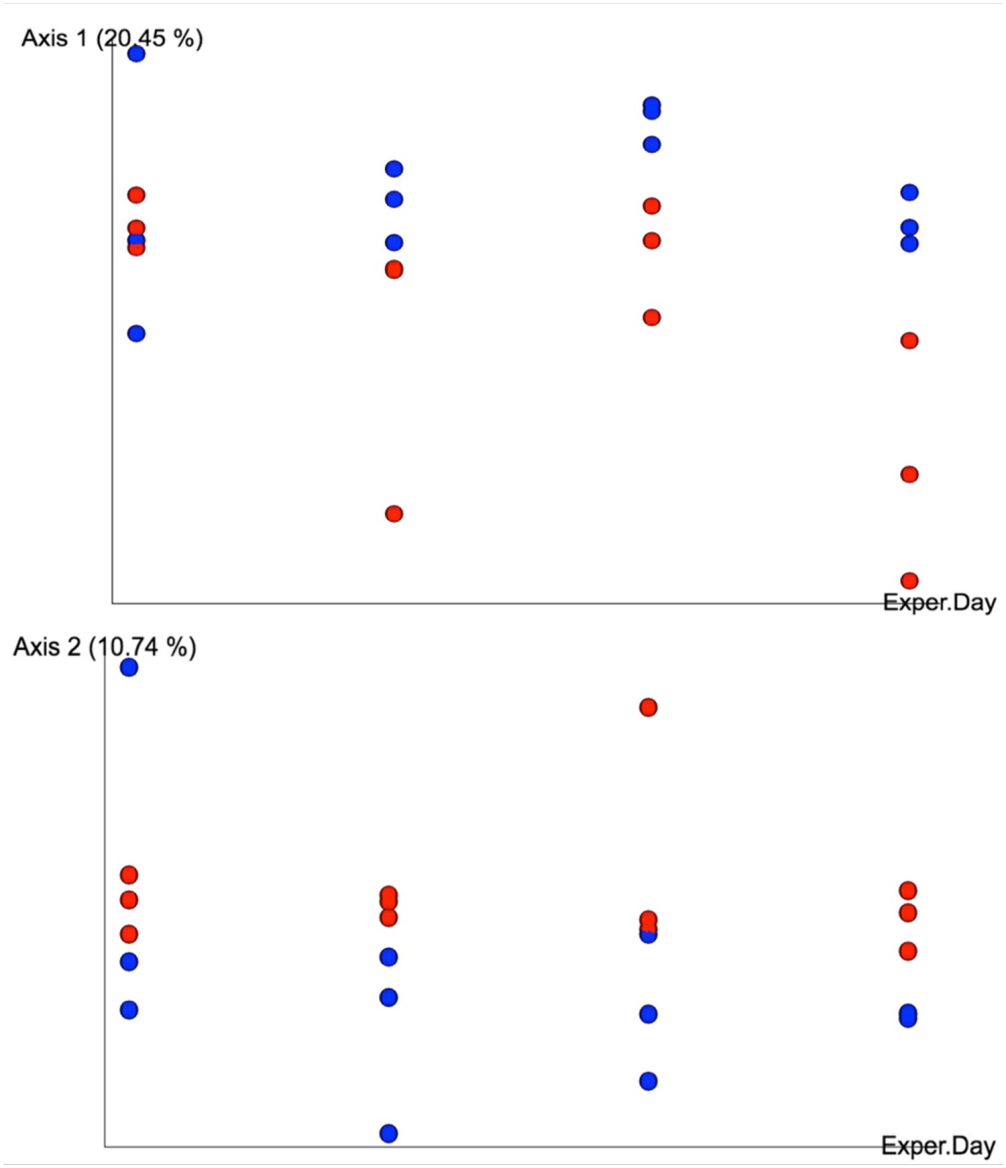
Principal Coordinates Analysis (PCoA) with weighted UniFrac distances. Red dots represent pH 7.6 samples and blue dots represent pH 8.0 samples from day 14–20 of a 21-day experiment. The horizontal axis is experiment day.

Given that all three alpha diversity measures significantly differed between pH treatments only during the final week of the experiment, beta diversity analyses focused on the final week of the experiment. The weighted UniFrac distances of samples between pH treatments was significantly greater than those of samples within pH treatment (PERMANOVA *pseudo-F* = 3.558; *p* < 0.01). During the final week of the experiment, samples from different pH treatments appeared to diverge along axis 1 in the PCoA plot, which accounted for 20.45% of community dissimilarity between treatments, with acidified samples showing greater within-group variation than the control samples (Fig 5). Samples between pH treatments also showed separation along axis 2, which accounted for 10.74% of the community dissimilarity between treatments (Fig 5). The ANCOM analysis revealed which taxa were responsible for driving the variation observed in the PCoA plot (see below).

### Taxa that differed in abundance across pH treatments with ANCOM

**Fig 6:**
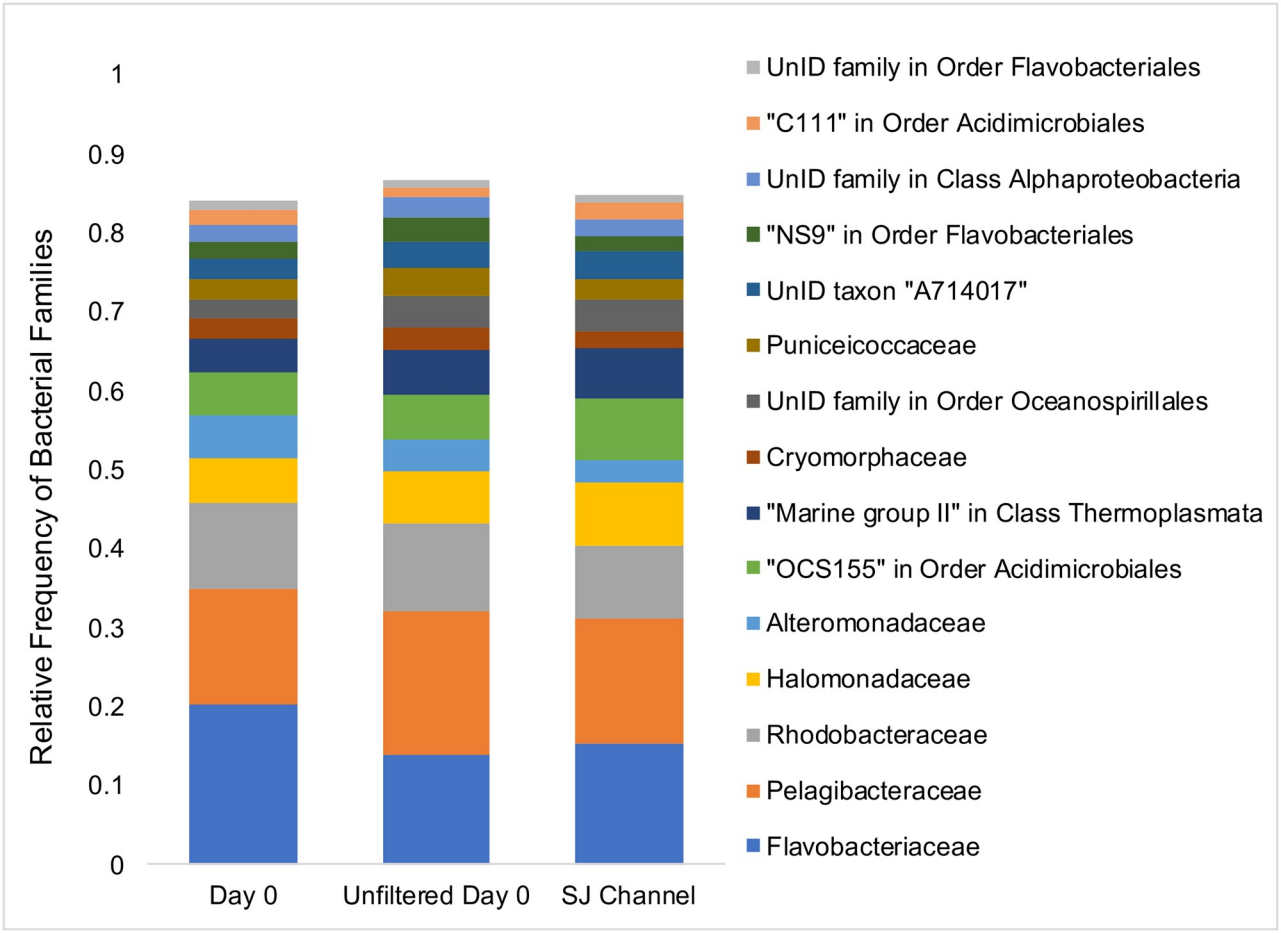
The relative frequency of the 15 most dominant bacterial families present in three seawater samples. The seawater samples include day 0 of the mesocosm experiment, both filtered and unfiltered, and San Juan Channel, directly offshore from the Ocean Acidification laboratory.

The relative abundance of the 15 most dominant bacterial families was very similar among the day 0 filtered seawater sample, the day 0 unfiltered seawater sample, and the San Juan Channel seawater sample collected offshore from the Friday Harbor Marine Laboratory (Fig 6). The relative frequencies of all detected microbial taxa, to the level of family, are provided from these three samples as well as all six mesocosm samples from day 20 (S1 Table).

During the final stage of the experiment (day 14–20), the ANCOM analysis revealed that acidification significantly decreased the relative frequency of four bacterial ASVs and it significantly increased the relative frequency of two bacterial ASVs (Table 1). The bacterial taxa that decreased under acidification are three members of the family Flavobacteriales (*Polaribacter dokodensis*, *Kordia jejudonensis*, *and Nonlabens marinus*) and one member of the family Hyphomonadaceae, (*Algimonas arctica*); centered log ratio mean differences [clr] between pH treatments were –6.65, -4.88, -3.79, and –3.77 respectively; Table 1). The bacterial taxa that increased under acidification are an unidentified species from the family Alteromonadaceae (clr = 3.85), and an ASV identified as *Polaribacter dokdonensis* (clr = 4.07), which has a 16S rRNA sequence that is 99% identical (420 out of 422 bp) to an ASV that decreased under acidification, also identified as *Polaribacter dokdonensis* (Table 1).

**Table 1:** ANCOM (Analysis of Composition of Microbiomes) results showing ASVs with significantly different abundances in pH 8.0 vs. pH 7.6 treatments during day 14–20 of a 21-day experiment.

Taxonomic ID of each ASV	Blast Score	Query cover	E value	% ID	W	clr	Median ASV count pH 8.0	Median ASV count pH 7.6	Max ASV count pH 8.0	Max ASV count pH 7.6
Flavobacteriaceae: Polaribacter dokdonensis*	769	100%	0	99.5	1144	-6.65	1656	0	40467	41
Flavobacteriaceae: Kordia jejudonensis*	769	100%	0	99.5	1094	-4.88	374	0	3020	523
Hyphomonadaceae: *Algimonas arctica*	732	100%	0	99.5	1017	-3.77	87	0	444	0
Flavobacteriaceae: Nonlabens marinus*	713	100%	0	97.2	948	-3.79	110	0	931	340
Flavobacteriaceae: Polaribacter dokdonensis*	769	100%	0	99.5	1074	4.07	0	48	0	357
Alteromonadaceae: *HTCC2207*	NA	NA	NA	NA	945	3.85	0	80	0	837

Gray coloration denotes a decrease in abundance in the acidic treatment compared to the control and white coloration denotes the opposite pattern. NCBI blast score, query cover, E-value, and % ID are shown for taxa names that have an asterisk where “NA” means that only the Greengenes 13_8 99% OTUs database was used for taxonomic identification. The ANCOM “W” statistic is the number of instances where the ratio of the frequency of a given ASV to the frequency of another ASV was significantly different between pH treatments out of 1,145 ASVs total. The ANCOM “clr” corresponds to the “centered log ratio” mean difference between pH treatments where the ratio is the frequency of a given ASV to the frequency of another ASV in a sample. A negative clr indicates a decrease in the frequency of an ASV in pH 7.6 relative to pH 8.0. The median ASV count (the 50^th^ percentile) and maximum ASV count from raw data for all of the samples within a given pH treatment are shown. The first and fifth taxa are both identified as *Polaribacter dorkodensis* and their 16S rRNA sequences are 99% identical (420 out of 422 bp).

## Discussion

### Decrease in microbial diversity under acidification

A decrease in microbial species richness and evenness under acidification rejects the null hypothesis of no response to OA from the microbial community in the Salish Sea, a region of naturally high pCO_2_ and low pH. Some recent mesocosm studies have observed shifts in bacterial community composition in response to OA [13, 17, 45, 46] but to my knowledge, this is the first mesocosm study of marine microbes to report an effect of OA on both microbial species richness and evenness. The prediction that OA would not affect microbial species richness and evenness in this region was based on the hypothesis that microbial species would already be adapted to a wide range of pH conditions due to regular, natural fluctuations in pH [23]. Such fluctuation might result in (1) elevated physiological plasticity [47] or (2) high standing genetic diversity [48] with respect to cell structures and processes associated with carbon fixation, carbon concentration, and pH homeostasis. This may not be true in this microbial community given that species richness and evenness declined under acidified conditions. Perhaps a significant portion of the bacterial community is dormant or inactive at any given time [49–51] and species that are well adapted to the ambient pCO_2_ / pH conditions comprise the active portion of the community. Evidence from a deep sequencing study, suggests that there is a persistent microbial seed bank in the world’s oceans and that geographic differences in microbial community structure are a result of shifts in the relative abundance of taxa rather than their presence or absence [52].

Given that microorganisms larger than 2.7 μm (including grazers of picoplankton) were excluded from the mesocosms, changes in grazer abundance can be eliminated as a potential cause for the observed changes in picoplankton community composition, richness, and evenness. However, the physiological cause(s) for the observed decline in species richness and evenness are unknown. It can only be inferred that fewer picoplankton species thrive in a pH 7.6 environment compared to a pH 8.0 environment. The ANCOM results from this study provided some prospects identifying culturable microbial species to examine in laboratory experiments that explore how acidification affects metabolic processes and/or population growth rate.

Three of the four bacterial species that decreased under acidification belonged to the family Flavobacteraceae, including *Polaribacter dokdonensis*, *Kordia jejudonensis*, and *Nonlabens marinus*. This is, perhaps, not surprising given that Flavobacteriaceae are often the numerically dominant bacterial family in marine environments, particularly in polar and near polar regions and among surface-associated bacteria. Flavobacteriaceae is the largest family in the phylum Bacteroidetes containing at least 90 genera and hundreds of species, most of which are heterotrophic, but vary significantly in their physiology and ecology [53, 54]. Flavobacteriaceae have been observed to decrease under acidification in a coastal sediment mesocosm study [13] and in a seawater mesocosm study [17]. Moreover, Flavobacteriaceae was reported to be the family with the highest contribution to pH-dependent dissimilarities across pH treatments in a mesocosm study but their response to acidification was dependent on season; Flavobacteriaceae decreased under acidification in the fall and winter but increased in the spring with no trend in the summer [17]. It would be interesting to replicate this study during different seasons. Given that the ambient pH in this region tends to be more acidic during the fall and winter [23, 30], the microbial community may show seasonal adaptation to pH and show no reduction in species richness or evenness with experimental acidification during these seasons.

The ASV that showed the biggest decline under acidification had a 16S rRNA sequence that was 99% identical to the ASV that showed the biggest increase under acidification and both were identified as *Polaribacter dokdonensis* (family Flavobacteriaceae). There appears to be sub-OTU (operational taxonomic unit) specialization to different pH environments for *P*. *dokdonensis*. Sub-OTU habitat specialization has been observed in marine bacteria with respect to season abundance, including members of Flavobacteriaceae [55]. Cyanobacteria strains with less than 3% difference in their 16S rRNA sequence showed niche partitioning by temperature, light, nutrients, and competitor abundance (Johnson et al. 2006). The type strain of *Polaribacter dokdonensis* has been cultured in the lab with an optimum pH of 7.0–8.0 [56]. It would be worthwhile to isolate each of the *P*. *dokdonensis* strains, one that is adapted to pH 7.6 and one that is adapted to pH 8.0, and compare their gene expression and cellular physiology in different pH environments. The other bacteria that decreased under acidification, *Kordia jejudonensis*, *Nonlabens marinus* (Flavobacteriaceae), and *Algimonas arctica* (Hyphomonadaceae) could also be examined in laboratory OA experiments, as they have been cultured with optimum pH ranges of 7–8, 5–10, and 7–8 respectively [57–59].

## Conclusion

In a large-scale review, bacterial community composition was more often found to be sensitive to various disturbances rather than resistant and this was true in 60% of studies with changes in CO_2_, albeit most studies examined soil communities rather than marine communities [60]. The observed decrease in microbial community richness and evenness under acidification in a region that regularly experiences high pCO_2_ and low pH suggests that such a community is still sensitive to acidification. A reduction in certain heterotrophic bacteria, mostly Flavobacteriaceae, which are known for utilizing proteins and polysaccharides, could affect the efficiency of the microbial food web, which would affect the availability of energy to higher trophic levels [61]. However, the reduction in community richness and evenness may or may not have an effect on functional diversity in the microbial community of the Salish Sea because different microbial taxa can carry out the same function in an ecosystem (functional redundancy) [60]. Additional mesocosm studies are needed that combine marker gene profiling with meta-transcriptomics and/or metametabolomics [22, 62] to help elucidate how OA affects marine microbial community processes such as respiration, photosynthesis, nitrogen fixation, organic matter degradation, and trophic interactions. This, in turn, will allow us to make more accurate predictions about how OA will affect the marine food web, marine biogeochemical cycles, and the ocean’s ecosystem services.

## Supporting information

S1 File Protocol for extracting DNA from seawater using Sterivex filter capsules. (PDF)

S1 Fig Three different measures of alpha diversity vs. sequencing depth including Shannon’s diversity index, Faith’s phylogenetic distance, and observed ASVs. (TIF)

S1 Table Relative frequencies of all detected microbial taxa at the level of family from seawater samples. The samples include: day 0 filtered seawater sample, day 0 unfiltered seawater sample, San Juan Channel seawater sample, and day 20 mesocosm samples A-F, where A-C are control mesocosms (pH 8.0) and D-F are acidified mesocosms (pH 7.6).
(XLSX)
