# Supplementary material for: Acidification decreases microbial community diversity in the Salish Sea, a region with naturally high pCO2
Source: PLoS One. 2020 Oct 28;15(10):e0241183. doi: 10.1371/journal.pone.0241183 (PMC7592811; doi:10.1371/journal.pone.0241183)
Supplement: S1 File — (PDF) [file pone.0241183.s001.pdf]

Protocol: DNA extraction from seawater using Sterivex filter capsules.

Modified by Lisa Crummett from Celine Mouginot's protocol developed in Adam Martiny's lab

1. After filtration, remove seawater from Sterivex filter capsule using a 30 mL syringe. Seal the smaller end of the Sterivex filter capsule with Crito-seal.
2. Add 1620 µL lysis buffer into each Sterivex filter capsule. Seal off the larger end of the capsule by attaching a luer lock 3 mL syringe.
3. Freeze at -20°C until extraction.

START EXTRACTION (DAY 1)

4. Defrost Sterivex filter capsules
5. Make fresh lysozyme solution (50 mg/mL) for each Sterivex filter

    For 1 Sterivex: 10 mg lysozyme (*Sigma Aldrich L1667-1G Human Lysozyme*) + 200 µL lysis buffer (see recipe below) into 2 mL centrifuge tube; invert multiple times and vortex lightly to dissolve the lysozyme powder. Make sure all the lysozyme is dissolved in lysis buffer before proceeding.
6. Add 180 µL lysozyme per sterivex and vortex lightly by rolling the filter over the vortexer.
7. Incubate sterivex filter capsule at 37°C for 30 min
8. Add 180 µL Proteinase K (1 mg/mL). Proteinase K can be kept at -20°C for 6 months
9. Add 100 µL 10% SDS per Sterivex filter capsule; invert multiple times and vortex lightly by rolling the filter on the vortexer.
10. **10.** Incubate Sterivex filter capsules at 55°C **overnight** on a shaker inside an incubator.

    Capsules should be kept upright (syringe-end pointing down and Crito-seal-end pointing up) and in constant motion on a plate shaker

STOP - OVERNIGHT

11. Pull solution into 3 mL syringe. First, remove the syringe from the Sterivex filter capsule. Pull back the plunger a little to add some air inside the syringe. Reattach the syringe to the Sterivex filter capsule and invert the capsule several times. Let the bubbles flow towards the top. Start pulling slowly on the plunger until you cannot pull back anymore. Remove the capsule from the syringe to get the maximum amount of liquid in the syringe.
12. Put solution (~2 mL) into a 5 mL centrifuge tube
13. Rinse the Sterivex filter capsule with 1000 µL TE buffer, inverting multiple times and vortexing gently
14. Add the TE in the Sterivex filter capsule to the 5 mL centrifuge tube and quickly vortex (2-3 sec)
15. Divide the liquid between four 1.5-mL centrifuge tubes, each tube should contain ~ 750 mL liquid
16. Add 125 µL of Na acetate to each tube
17. Add 495 µL of isopropanol to each tube; invert multiples times
18. Incubate tubes at -20°C for **> 2 hours**

STOP - 2 hours

CENTRIFUGE NEEDS TO BE AT 4°C FOR STEP 19

19. Centrifuge tubes at 15,000 xg (rcf) for 30 min at 4°C
20. Remove supernatant (gently dump out all liquid waste) and add 100 µL of TE buffer into each tube; vortex lightly
21. Incubate tubes at 37°C for **30 – 60 min**
22. Centrifuge tubes for a few seconds at 10,000 xg to pull down condensation. Then, pool the solution from the four tubes (from a single Sterivex filter capsule) into one 1.5 mL centrifuge tube.

THE FOLLOWING STEPS USE THE ZYMO DNA CLEAN & CONCENTRATOR KIT

15. Add 1200 µL DNA binding buffer to each tube, invert several times to mix

16. Add 950 µL of solution to a spin column, and let stand for 1 min
17. Centrifuge at 12,000 xg for 30 sec
18. Discard flow-through
19. Add remaining solution to the column and repeat steps 17 – 18
20. Add 200 µL Wash Buffer to the column - MAKE SURE THE WASH BUFFER HAS 100% ETHANOL ADDED
21. Centrifuge at 12,000 xg for 1 min
22. Repeat steps 20 - 21
23. Move column to a clean and well labeled 1.5 mL Eppendorf tube
24. Add 25 µL TE buffer to column, let stand for 1 min
25. Centrifuge at 12,000 xg for 30 sec
26. Repeat steps 24 - 25
27. Store at -20°C

Required Solutions:

- To make 100 mL of **Tris HCl 1M**:
  In a 100 mL volumetric flask with 50 mL of autoclaved milliQ (ultrapure) water, add 12.11 g of Tris base (mw 121.14g/mol). Dissolve and add HCl drops to reduce the pH to 7.6. Add milliQ water to bring the volume to 100 mL. Store at room temperature.

- To make 250 mL of **Lysis Buffer**:
  In a 250 mL volumetric flask with 150 mL of autoclaved milliQ water, add 5.84 g of NaCl (mw 58.44g/mol), 64.18 g of sucrose (mw 342.3 g/mol), 12.5 mL of Tris HCl 1M and 10 mL of EDTA 0.5M.
  Dissolve and bring volume to 250ml. Then autoclave. Let it cool down and store in the fridge.

- To make 500 mL of **TE Buffer**:
  In a 500 mL volumetric flask with 400 mL of autoclaved milliQ water, add 5 mL of 1M Tris pH-8 and 1 mL of 0.5M EDTA. Mix and bring volume to 500 mL. Make aliquots and autoclave. Store at room temperature.

- To make 10 mL of **Proteinase K**:
  In a 50 mL falcon tube, add 50 mg of Proteinase K powder. Bring volume to 50 mL. Mix and make aliquots. Store in -20°C freezer.

- To make 50 mL of **Na Acetate**:
  In a 50 mL bottle with 20 mL of autoclaved milliQ water, add 12.25 g of Na Acetate (mw 82.03 g/mol). Add acetic acid to reduce the pH to 5.2. Measure the volume and bring the final volume to 50 mL with milliQ water. Autoclave and store at room temperature.
