# Supplementary figures and images for: Acidification decreases microbial community diversity in the Salish Sea, a region with naturally high pCO2
Source: PLoS One. 2020 Oct 28;15(10):e0241183. doi: 10.1371/journal.pone.0241183 (PMC7592811; doi:10.1371/journal.pone.0241183)

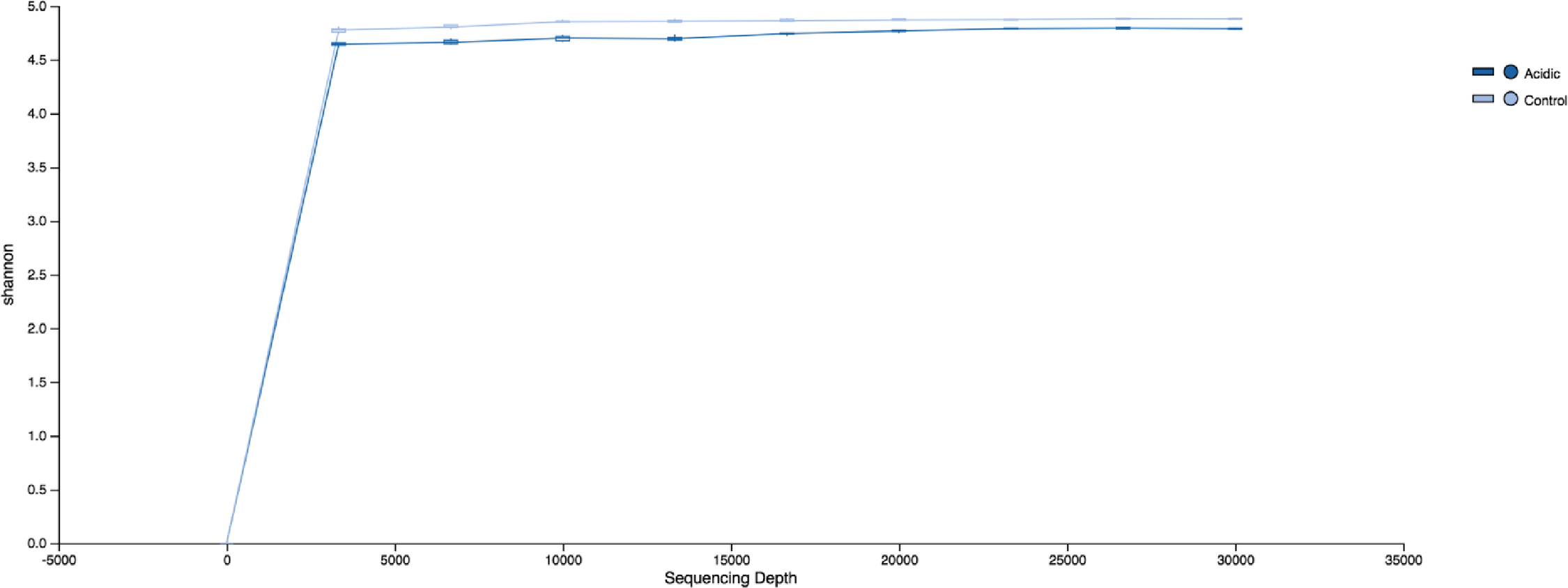

Supplement: S1 Fig — (TIF) [file pone.0241183.s002.tif]
